# Stand-Alone Lateral Lumbar Interbody Fusion at L3-L4 with 3D-Printed Porous Titanium Cages: A Safe and Effective Alternative in the Treatment of Degenerative Disc Disease (DDD)

**DOI:** 10.3390/jcm14124233

**Published:** 2025-06-14

**Authors:** Luca Ricciardi, Andrea Perna, Sokol Trungu, Massimo Miscusi, Alba Scerrati, Annamaria Narciso, Salvatore Cracchiolo, Sara Favarato, Antonino Raco

**Affiliations:** 1 UOC di Neurochirurgia, Azienda Ospedaliera Universitaria Sant’Andrea, Dipartimento NESMOS, Sapienza Università di Roma, 00185 Rome, Italy; sokol.trungu@gmail.com (S.T.); narciso.1922411@studenti.uniroma1.it (A.N.); antonino.raco@uniroma1.it (A.R.); 2 Department of Orthopedics and Trauma Surgery, Fondazione Casa Sollievo Della Sofferenza IRCCS, 71013 San Giovanni Rotondo, Italy; perna.andrea@gmail.com; 3 UOC di Neurochirurgia, Aziendal Ospedaliera Sant’Anna, Dipartimento di Medicina Traslazionale e per la Romagna, Università degli Studi di Ferrara, 44122 Ferrara, Italy; massimo.miscusi@unife.it (M.M.); scrlba@unife.it (A.S.); salvatore.cracchiolo@edu.unife.it (S.C.); sara.favarato@edu.unife.it (S.F.)

**Keywords:** spine, minimally invasive spine surgery, fusion, degenerative disc disease, LLIF, low back pain, intervertebral disc

## Abstract

**Background/Objectives:** Stand-alone lateral lumbar interbody fusion (LLIF) remains a debated approach in spinal surgery, with limited published evidence supporting its efficacy without supplemental fixation. This prospective study presents the institutional case series on single-level L3-L4 stand-alone LLIF, using next-generation 3D-printed titanium cages, as treatment for degenerative disc disease (DDD). **Methods:** A cohort of 49 patients with symptomatic DDD, unresponsive to conservative therapy, underwent stand-alone LLIF at L3-L4 (neither posterior pedicle screws nor lateral plating). Clinical outcomes (VAS and ODI) and radiological parameters (disc height, segmental/lumbar lordosis) were collected preoperatively and at 1, 6, and 12 months. Repeated-measures ANOVA with Bonferroni correction was adopted for statistical analysis. **Results:** Significant improvements were observed in pain and disability scores at all time points, with the mean VAS score decreasing from 6.53 to 0.29, and ODI from 27.6% to 3.84% at one year (*p* < 0.001). Radiographic analysis confirmed durable increases in disc height and segmental lordosis. Solid fusion was achieved in 97.9% of cases. No patient required posterior revision; transient neurological symptoms were mild and self-limiting. **Conclusions:** This study demonstrates that stand-alone LLIF at L3-L4 is safe and effective in achieving stable fusion and clinical–radiological improvement. These results challenge the necessity of supplemental fixation and support the broader adoption of a less invasive fusion paradigm.

## 1. Introduction

Degenerative disc disease (DDD) is a progressive condition primarily affecting the intervertebral discs, with the lumbar spine being particularly vulnerable due to its biomechanical load-bearing function [1]. Among the most recognized etiopathogenetic theories is the imbalance between anabolic and catabolic processes in the nucleus pulposus (NP), leading to a depletion of its extracellular matrix and biomechanical failure [2]. However, alternative mechanisms such as repetitive microtrauma, altered spinal kinematics, and chronic low-grade infections may act independently or synergistically, accelerating the degenerative cascade [2,3].

Clinically, DDD typically manifests as mechanical low back pain, often exacerbated by upright posture, forward flexion, and axial loading. Radiological diagnosis relies heavily on magnetic resonance imaging (MRI), where disc degeneration is staged using the Pfirrmann grading system, often accompanied by Modic changes at adjacent vertebral endplates [4,5]. Advanced degeneration may lead to disc herniation, facet overload, and ligamentous hypertrophy, potentially resulting in segmental stenosis and instability. Dynamic radiographs are complementary in assessing segmental mobility, sagittal alignment, and instability patterns [6].

Since DDD intrinsically affects the discal compartment, surgical interventions should prioritize restoring disc integrity while preserving posterior elements, including paraspinal musculature and facet joints. Minimally invasive anterior, oblique, and lateral lumbar interbody fusion (LLIF) techniques offer direct access to the disc with minimal-to-absent posterior disruption [7]. LLIF, particularly in the form described by Pimenta et al. [8], utilizes a retroperitoneal transpsoas corridor, requiring meticulous neuromonitoring to limit lumbar plexus injury risks. Although associated with specific complications—including transient neurological deficits, abdominal wall hypotonia, and vascular or ureteric injury—the technique allows the positioning of large-footprint cages spanning the entire disc space, then optimizing disc height restoration, coronal alignment, and segmental lordosis [8].

Interbody cage insertion through LLIF aims at segmental stabilization (primary synthesis) and osseointegration (secondary fusion) across the disc space. Traditionally, supplemental fixation via lateral plating or posterior pedicle screws has been employed to enhance construct stability [9]. However, stand-alone LLIF has increasingly gained acceptance, especially in managing adult spinal deformity (ASD) and adjacent segment degeneration, showing promising results in terms of sagittal alignment and clinical improvement while reducing operative morbidity [9].

This study aims to evaluate the clinical and radiological outcomes and perioperative complications associated with stand-alone LLIF in patients with single-level lumbar DDD, treated at three institutional centers under standardized protocols.

## 2. Materials and Methods

### 2.1. Study Design and Ethical Compliance

This prospective observational case series was conducted according to the CARE guidelines [10] across three spine care centers. Institutional ethics committee approval was waived due to the non-interventional nature of the study and adherence to standard treatment protocols. All procedures conformed to the ethical standards set by the 1964 Helsinki Declaration and its amendments. All patients provided written informed consent for the surgical procedure and anonymized data collection for research purposes.

### 2.2. Patient Selection

From January 2021 to December 2023, consecutive patients presenting with chronic (>3 months) low back pain refractory to conservative management were screened. Inclusion criteria comprised MRI-confirmed Pfirrmann grade [4,5] 4 or 5 degeneration at L3-L4, with or without Modic type I changes, reduced disc height or segmental lordosis on lateral X-rays, and low-grade spondylolisthesis (Meyerding grade I) [11] without dynamic instability (defined as slippage >3 mm on flexion-extension radiograms) [12]. Patients were excluded if they had previous lumbar surgery, systemic oncological disease, radiological evidence of multi-level degeneration, T-score < −2.5 on DEXA, or if intraoperative implant subsidence (>2 mm) necessitated posterior screw supplementation [13].

### 2.3. Surgical Technique

All procedures were performed by experienced spine surgeons (L.R., M.M., A.R.), each certified for the XLIF technique. A left-sided retroperitoneal transpsoas approach was uniformly adopted. The interbody device was a 3D-printed titanium cage (Modulus, Globus Medical, Audubon, PA, USA) with 10° of intrinsic lordosis, filled with osteoinductive putty (Attrax, Globus Medical, USA). Cage length and footprint were tailored to the patient’s anatomy, ensuring 1–2 mm lateral overhang on AP view for optimal endplate coverage. No supplemental lateral plating or posterior pedicle screws were employed in any case.

Postoperative drainage was placed in all cases and removed within 24 h. All patients underwent standardized post-operative care, mobilization, and rehabilitation protocols.

### 2.4. Outcome Assessment

Demographic and clinical data collected at baseline included age, sex, BMI, smoking status, comorbidities, and symptom duration. Pain intensity was assessed using a 10-point Visual Analogue Scale for back pain (VAS-b), and functional disability using the Oswestry Disability Index (ODI). Radiological parameters included Pfirrmann grade, Modic changes, disc height, and sagittal alignment (segmental L3-L4 and global lumbar lordosis).

Post-operative radiographs were obtained before discharge, with follow-up imaging at 1 and 12 months (including dynamic views at 12 months to assess for fusion). Successful fusion was defined radiographically as the absence of movement on flexion-extension radiographs [8,14].

### 2.5. Statistical Analysis

All data were collected prospectively and retrospectively reviewed. Continuous variables are presented as mean ± standard deviation (SD). Normality of distributions was assessed using the Shapiro–Wilk test. Categorical variables are reported as counts and percentages. To evaluate clinical outcomes over time (VAS-back and ODI scores), a one-way repeated-measures ANOVA was performed when the assumptions of normality were met. The Friedman test was used as a non-parametric alternative for non-normally distributed data. Bonferroni correction was applied for multiple pairwise comparisons. Effect sizes (Cohen’s d) were calculated to assess the clinical relevance of outcome changes, with standard thresholds of 0.2 (small), 0.5 (moderate), and 0.8 (large) applied.

Radiological outcomes (disc height, segmental and global lumbar lordosis) were evaluated using paired *t*-tests or the Wilcoxon signed-rank test depending on data distribution. Fusion rates at 12 months were assessed via dynamic radiographs and defined as the absence of motion at the treated level. Predictive analyses for postoperative complications and transient neurologic symptoms (e.g., BMI > 30, smoking, psoas retraction time) were performed using univariate logistic regression for categorical variables and independent samples *t*-tests for continuous variables. Multivariate regression was not performed due to the limited number of outcome events.

A post hoc power analysis was conducted to verify whether the study sample was sufficient to detect clinically meaningful differences in VAS and ODI scores over time. With a sample size of 49, alpha of 0.05, and a large effect size (Cohen’s d = 0.8), the statistical power exceeded 90% for the primary clinical endpoints, confirming adequate sensitivity to detect significant changes. All analyses were performed using StatPlus Pro v8 (AnalystSoft Inc., Walnut, CA, USA). A two-sided *p*-value < 0.05 was considered statistically significant.

## 3. Results

### 3.1. Patient Cohort

A total of 49 patients (26 females and 23 males; mean age 58.2 ± 10.7 years; mean BMI 27.6 ± 3.4 kg/m^2^) were included in the study. Of these, 34.6% were active smokers. The average duration of symptoms before surgery was 4.51 ± 1.31 months. Baseline clinical assessment revealed a mean Visual Analog Scale (VAS) for back pain of 6.53 ± 0.74 and a mean Oswestry Disability Index (ODI) of 27.60% ± 7.27%. According to Pfirrmann grading, disc degeneration was classified as grade III in 63% of cases, grade IV in 35%, and grade V in 2%, while Modic type I changes were present in 43% of patients (see Table 1).

### 3.2. Surgical Data and Complications

All surgeries were conducted via a left-sided XLIF approach using a 3D-printed porous titanium cage with integrated 10° lordosis, filled with Attrax Putty (Modulus^®^, Globus Medical). The mean psoas retraction time was 22.7 ± 9.4 min, and the average operative time was 48 ± 11.3 min. Estimated intraoperative blood loss remained under 100 mL in all cases; no perioperative transfusions were necessary. Surgical drains were consistently removed within 24 h postoperatively. Seven patients (14%) experienced transient paresthesia in the left L4 dermatome, and three (6%) reported transient quadriceps weakness. All symptoms resolved spontaneously within two weeks. There were no cases of persistent neurological deficit, postoperative ileus, ureteral injury, or deep infection. Exploratory univariate analysis of potential predictors of transient neurologic symptoms, including BMI >30, smoking status, and psoas retraction time, revealed no statistically significant associations (*p* > 0.05). However, a non-significant trend was noted between longer retraction times (>25 min) and transient paresthesia (*p* = 0.09) (see Table 2).

### 3.3. Clinical and Functional Outcomes

**Figure 1:**
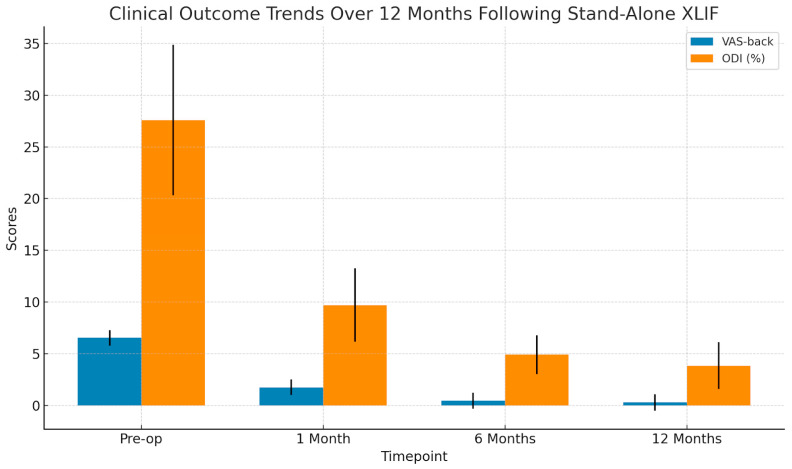
Changes in Visual Analogue Scale (VAS-back) and Oswestry Disability Index (ODI) scores across the follow-up period. Both clinical parameters show a marked and statistically significant improvement after surgery. The VAS-back score decreased from a baseline of 6.53 to 0.29 at 12 months, while ODI improved from 27.6% to 3.84%, highlighting the effectiveness of stand-alone XLIF in pain relief and functional recovery. Error bars represent standard deviation.

Clinical outcomes improved markedly over time. Repeated-measures ANOVA revealed statistically significant reductions in VAS and ODI scores across all follow-up intervals (*p* < 0.001 for both measures). The mean VAS decreased from 6.53 ± 0.74 preoperatively to 1.74 ± 0.76 at one month, 0.47 ± 0.78 at six months, and 0.29 ± 0.79 at one year. ODI showed a similar trajectory, improving from 27.60% ± 7.27% at baseline to 9.71% ± 3.54% at one month, 4.90% ± 1.87% at six months, and 3.84% ± 2.27% at one year (see Figure 1).

Pairwise comparisons confirmed statistically significant improvements at each time point (Bonferroni-adjusted *p* < 0.01), with large to very large effect sizes observed (Cohen’s d > 2.5) (see Table 3).

### 3.4. Radiographic Results

**Figure 2:**
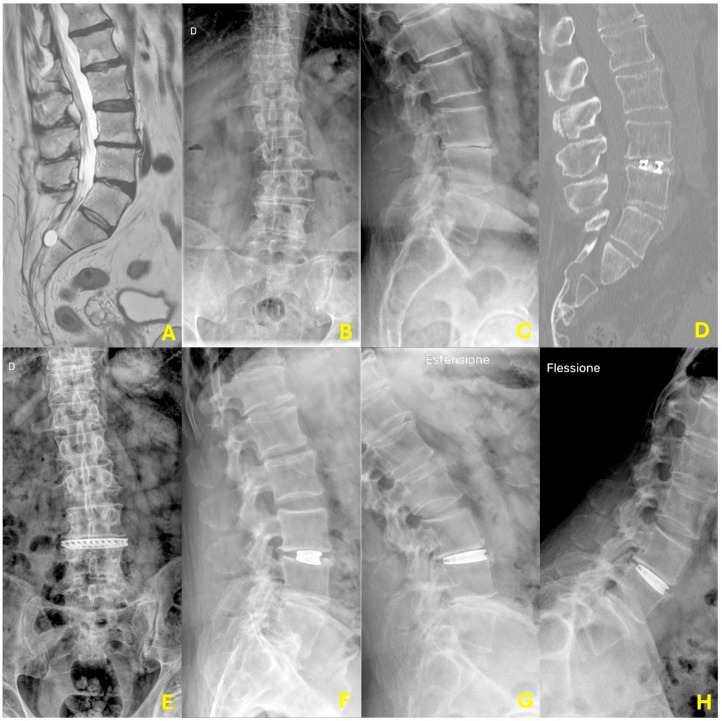
Case presentation. This is the case of a 66-year-old gentleman, suffering from Pfirrman grade IV degenerative disc disease with Modic change at L3-L4 (**A**), significantly reduced disc height and segmental lordosis (**B**,**C**), and 3 mm lateral listhesis of L3 on L4 on the convexity side. The postoperative CT scan (**D**) and X-rays (**E**,**F**) show the implanted 3D printed porous titanium cage correctly positioned at L3-L4, restoring the disc height and the segmental lordosis without subsidence in the inferior endplate. In the one-year dynamic x-rays (**G**,**H**), there is no evidence of residual segmental movement or subsidence, while the segmental alignment and disc height are maintained.

Radiographic measurements demonstrated substantial initial gains that partially regressed over time, without losing statistical or clinical relevance. Mean disc height increased significantly from 6.37 ± 1.34 mm preoperatively to 10.04 ± 1.34 mm at one month (*p* < 0.01), followed by slight reductions to 9.65 ± 1.18 mm at six months (*p* = 0.12) and 9.33 ± 1.21 mm at twelve months (*p* = 0.19), remaining significantly improved from baseline. Segmental lordosis at L3-L4 improved from a preoperative value of 6.67 ± 1.63° to 10.69 ± 1.79° at one month (*p* < 0.01), then declined slightly to 10.04 ± 1.37° at six months (*p* = 0.05), and to 9.88 ± 1.36° at one year (*p* = 0.56), maintaining a clinically beneficial correction. Similarly, global lumbar lordosis increased from 57.25 ± 7.31° to 60.27 ± 5.5° at one month (*p* = 0.02), reached 61.31 ± 4.83° at six months (*p* = 0.32), and was sustained at 60.76 ± 4.63° at one year (*p* = 0.57). At final follow-up, radiographic evidence of fusion was confirmed in 48 out of 49 patients (97.9%) using flexion-extension radiographs. A single case of significant cage subsidence (>2 mm) was observed, necessitating a posterior instrumentation procedure (see Table 4). A case presentation is reported in Figure 2.

## 4. Discussion

### 4.1. Background and Rationale

Degenerative disc disease (DDD) of the lumbar spine remains one of the most prevalent causes of chronic low-back pain, mainly affecting middle-aged and older adults. The pathophysiology is multifactorial, although the loss of balance between matrix synthesis and degradation within the nucleus pulposus (NP) has been reported as a central mechanism, the role of other contributing factors, such as altered spinal biomechanics, chronic low-grade infections, genetic predisposition, and microtraumatic repetitive stress, should not be underestimated [2]. As the intervertebral disc progressively degenerates while aging, segmental instability, disc height collapse, facet joint overload, and subsequent neural element compression may lead to complex clinical syndromes that significantly impair the quality of life [2,15].

Historically, surgical treatment of DDD has aimed to alleviate symptoms through direct decompression and mechanical stabilization of the affected segments. Posterior approaches such as transforaminal or posterior lumbar interbody fusion (TLIF/PLIF) have been the most adopted surgical techniques, although related to inherent risks of iatrogenic injury to the paraspinal musculature, facet joints, and neural structures [16]. Over the past two decades, less-to-minimally invasive anterior and lateral access strategies have allowed surgeons to focus on disc space preparation and indirect decompression, eventually sparing the posterior column from iatrogenic surgical injury [8,16,17].

Lateral lumbar interbody fusion (LLIF), as preliminarily described by Pimenta and other authors, has become increasingly adopted in clinical practice due to its ability to restore disc height and segmental lordosis through a retroperitoneal transpsoas approach [8,17,18,19]. This technique allows for the implantation of interbody devices with a larger footprint than posterior cages, distributing the axial load more evenly across the vertebral endplates, enhancing primary stability, and promoting bony fusion [8,17,20]. The indirect decompressive effect, achieved by restoring the collapsed disc space and distracting the ligamentum flavum and foramina through a ligamentotaxis mechanism, is particularly advantageous in cases without significant posterior element hypertrophy or facet arthropathy [21,22].

However, the absolute necessity of supplemental fixation, either via lateral plating or posterior pedicle screw instrumentation, remains debated among surgeons [23]. Historically, stand-alone interbody cages were deemed insufficient to ensure long-term stability and fusion, particularly in osteopenic patients or multi-level disease [18,24,25,26,27]. The recent advent of 3D-printed porous titanium cages, characterized by an optimized elastic modulus closer to cancellous bone and a trabecular structure that promotes osseointegration, has challenged this paradigm [19,25,27]. These newer implants, filled with osteoinductive materials such as bioactive putties, may reduce the risk for subsidence and provide sufficient primary fixation to obviate the need for supplemental fixation in selected patients [19,23,24,27].

### 4.2. Analysis of Results and Clinical Implications

This prospective analysis supports the safety and effectiveness of single-level L3-L4 XLIF using a 3D-printed titanium cage filled with ceramic bone graft in carefully selected patients. Clinical outcomes demonstrated rapid and sustained improvements in pain and disability, with mean VAS and ODI scores showing statistically significant and clinically meaningful reductions at each follow-up interval. The risk of adjacent segment disorder after stand-alone XLIF was not evaluated in this paper, according to the relatively short follow-up time.

The dramatic decrease in VAS from a preoperative value of 6.53 to 0.29 at one year and a corresponding ODI improvement from 27.6% to 3.84% reflect the technical success and potential for early return to standard activity, then eventually reducing absence from work and health-related reduced quality of life. The magnitude of improvement, underscored by very large effect sizes, is consistent with prior literature on XLIF outcomes. Our findings stand out due to the rapid clinical recovery observed within the first postoperative month.

Radiographic parameters, including disc height and sagittal alignment, improved significantly postoperatively. The increase in disc height to 10.04 mm and segmental lordosis to 10.69° at one month indicates immediate and effective indirect decompression and sagittal plane correction. Although mild reductions in these values were observed over time, they did not reach statistical significance while remaining above baseline at one year, suggesting that some implant settling or biological remodeling occurred without compromising global alignment or clinical results. It is also relevant that the initial gain in disc height did not translate into increased subsidence rates, with only one patient requiring revision over the follow-up.

Segmental and global lordosis gains were modest but consistent with the implant device’s geometry, which featured 10° of lordosis. These values match the biomechanical expectations for lateral interbody fusion without supplemental posterior fixation. The maintenance of global lumbar lordosis above 60° postoperatively supports the role of XLIF in restoring spinal balance, particularly in patients with neutral or mildly hypolordotic spines.

The observed fusion rate of 97.9% at twelve months further validates the biological performance of the porous titanium cage and ceramic bone graft composite. This fusion rate compares favorably with traditional grafting materials and instrumentation strategies, suggesting modern biomaterials may reduce the need for additional autograft harvesting or posterior support in selected cases.

Neurological complications in this series were transient and resolved spontaneously. The 14% incidence of paresthesia and 6% of transient quadriceps weakness are within the expected range for the XLIF approach and likely related to intraoperative psoas manipulation. No permanent motor deficits were reported, and no major visceral or vascular injuries occurred, reinforcing the safety profile of the technique. Although no statistically significant predictors of transient complications were identified, the trend between prolonged psoas retraction and sensory disturbances warrants further investigation and may inform surgical planning and technique optimization.

### 4.3. Limitations

Our findings must be interpreted in light of several limitations. As a single-arm case series, the lack of a control group limits the ability to draw causal inferences. While adequate for a case series, the relatively small sample size reduces the power to detect rare adverse events or subtle subgroup differences. Our results may not be generalizable to L4-5, which is subjected to greater biomechanical loads and exhibits more complex kinematic behavior. Additionally, excluding osteoporotic patients limits applicability to older or more comorbid populations where supplemental fixation may still be indicated. Further randomized controlled trials, ideally stratified by level, bone quality, and preoperative segmental alignment, are necessary to establish the indications and limitations of stand-alone LLIF more definitively. Advanced imaging modalities and biomechanical modeling may also play a role in refining patient selection and predicting fusion outcomes based on implant-endplate interface characteristics. Lastly, the short follow-up limited the possibility to investigate the risk for adjacent segment disorder after stand-alone XLIF using 3D-printed porous titanium implants. Since the degeneration of the levels contiguous to the fused one may lead to second surgeries and increased health-related costs, this aspect should be carefully considered in the data interpretation.

## 5. Conclusions

Our study supports using stand-alone LLIF at L3-L4 as a safe, efficient, and clinically effective treatment for selected patients with low-back pain sustained by DDD. Incorporating modern implant technology and strict surgical indications may reduce invasiveness without compromising stability or fusion success. These findings contribute to the evolving landscape of minimally invasive spinal surgery and suggest a potential paradigm shift toward tailored, anatomy- and pathology-specific solutions in lumbar fusion surgery.

## Figures and Tables

**Table 1:** Patient demographics and baseline characteristics. VAS: Visual Analog Scale; ODI: Oswestry Disability Index; BMI: Body Mass Index.

Characteristic	Value
Total Patients	49
Sex	26 Female/23 Male
Mean Age (years)	58.2 ± 10.7
Mean BMI (kg/m^2^)	27.6 ± 3.4
Active Smokers	34.6%
Mean Symptom Duration (months)	4.51 ± 1.31
Baseline VAS (back pain)	6.53 ± 0.74
Baseline ODI (%)	27.60% ± 7.27%
Pfirrmann Grade III	63%
Pfirrmann Grade IV	35%
Pfirrmann Grade V	2%
Modic Type I Changes	43%

**Table 2:** Surgical data and complications. XLIF: Extreme Lateral Interbody Fusion. Sx: Symptoms.

Parameter	Details/Value
Surgical Approach	Left-sided XLIF
Implant Type	3D-printed porous titanium cage (Modulus^®^, Globus Medical), 10° lordosis
Graft Material	Attrax Putty
Mean Psoas Retraction Time (min)	22.7 ± 9.4
Mean Operative Time (min)	48 ± 11.3
Estimated Intraoperative Blood Loss	<100 mL (all cases)
Blood Transfusions Required	None
Surgical Drain Removal Time	Within 24 h postoperatively
Complications	
Transient Paresthesia (L4 dermatome)	7 patients (14%)
Transient Quadriceps Weakness	3 patients (6%)
Persistent Neurological Deficit	0 cases
Postoperative Ileus	0 cases
Ureteral Injury	0 cases
Deep Infection	0 cases
Significant Cage Subsidence (>2 mm)	1 case
Predictors of Transient Neuro. Sx	No statistically significant associations found (*p* > 0.05)

**Table 3:** Clinical and functional outcomes over time. VAS: Visual Analog Scale; ODI: Oswestry Disability Index. All follow-up values showed statistically significant improvement compared to baseline (pairwise comparisons, Bonferroni-adjusted *p* < 0.01).

Outcome Measure	Baseline	1 Month Post-Op	6 Months Post-Op	1 Year Post-Op	Overall Change (ANOVA)
Mean VAS (back pain)	6.53 ± 0.74	1.74 ± 0.76	0.47 ± 0.78	0.29 ± 0.79	*p* < 0.001
Mean ODI (%)	27.60% ± 7.27%	9.71% ± 3.54%	4.90% ± 1.87%	3.84% ± 2.27%	*p* < 0.001

**Table 4:** Radiographic outcomes over time. N/A: Not Applicable. * *p* < 0.01 compared to baseline. ^ *p* = 0.02 compared to baseline. ** Although slight reductions occurred after 1 month, values at 6 and 12 months remained significantly improved compared to baseline (inferred from text).

Radiographic Measure	Baseline	1 Month Post-Op	6 Months Post-Op	1 Year Post-Op	Change from Baseline at 1 Yr
Mean Disc Height (mm)	6.37 ± 1.34	10.04 ± 1.34 **	9.65 ± 1.18	9.33 ± 1.21	Significant *
Mean Segmental Lordosis L3-L4 (°)	6.67 ± 1.63	10.69 ± 1.79 **	10.04 ± 1.37	9.88 ± 1.36	Significant *
Mean Global Lumbar Lordosis (°)	57.25 ± 7.31	60.27 ± 5.5 ^	61.31 ± 4.83	60.76 ± 4.63	Significant *
Fusion Rate (at 1 year)	N/A	N/A	N/A	97.9% (48/49)	N/A

## Data Availability

The raw data supporting the conclusions of this article will be made available by the authors on request.

## Abbreviations

The following abbreviations are used in this manuscript:

DDD	Degenerative disc disease
XLIF	Extreme lateral interbody fusion
LLIF	Lateral lumbar interbody fusion
VAS	Visual Analogue Scale
ODI	Oswestry Disability Index
